# Coupling between the Basic Replicon and the *Kis-Kid* Maintenance System of Plasmid R1: Modulation by Kis Antitoxin Levels and Involvement in Control of Plasmid Replication

**DOI:** 10.3390/toxins7020478

**Published:** 2015-02-05

**Authors:** Juan López-Villarejo, Damián Lobato-Márquez, Ramón Díaz-Orejas

**Affiliations:** Department of Molecular Microbiology and Infection Biology, C/ Ramiro de Maéztu 9, Centro de Investigaciones Biológicas-CSIC, 28040 Madrid, Spain; E-Mails: villarejo@cib.csic.es (J.L.-V.); dami_lm@cib.csic.es (D.L.-M.)

**Keywords:** plasmid R1, plasmid replication and copy number control, *parD (kis-kid)* antitoxin-toxin system, Kis antitoxin, ClpAP protease, transcriptional regulation, CopB repressor, coupling plasmid maintenance systems

## Abstract

*kis-kid*, the auxiliary maintenance system of plasmid R1 and *copB*, the auxiliary copy number control gene of this plasmid, contribute to increase plasmid replication efficiency in cells with lower than average copy number. It is thought that Kis antitoxin levels decrease in these cells and that this acts as the switch that activates the Kid toxin; activated Kid toxin reduces *copB*-mRNA levels and this increases RepA levels that increases plasmid copy number. In support of this model we now report that: (i) the Kis antitoxin levels do decrease in cells containing a mini-R1 plasmid carrying a *repA* mutation that reduces plasmid copy number; (ii) *kid*-dependent replication rescue is abolished in cells in which the Kis antitoxin levels or the CopB levels are increased. Unexpectedly we found that this coordination significantly increases both the copy number of the *repA* mutant and of the wt mini-R1 plasmid. This indicates that the coordination between plasmid replication functions and *kis-kid* system contributes significantly to control plasmid R1 replication.

## 1. Introduction

Plasmid R1 is an antibiotic resistance plasmid of enteric bacteria that has contributed important insights into plasmid replication and its control as well as into the regulation and role of auxiliary plasmid maintenance systems [1]. R1 is maintained with a low copy number in the host. Its replication is initiated due to specific interactions of a rate limiting protein, RepA, at *oriR1*, the origin of replication [2]. The frequency of this process is regulated by the copy number control genes *copA* and *copB*. *copA*, the key regulator gene, codes an unstable antisense RNA, CopA, that inhibits at the posttranscriptional level the synthesis of RepA. CopA RNA targets the polycistronic *copB-repA* mRNA at *copT*, its complementary sequence, and inhibits translation of the *tap* orf which is needed for RepA translation [3,4]. Inactivation of *copA* leads to uncontrolled amplification of the plasmid or run-away replication [5].

**Figure 1:**
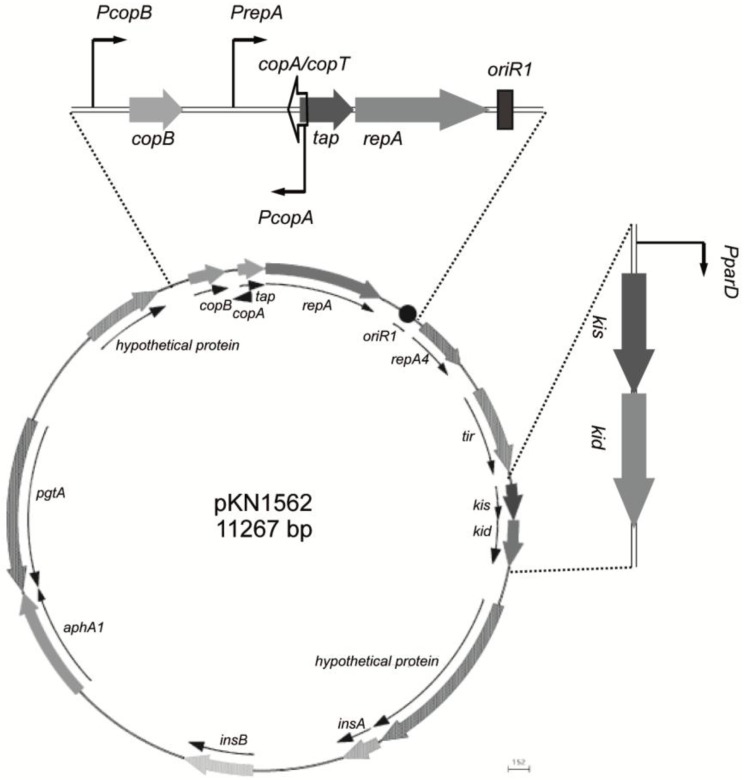
Localization of maintenance regions in the mini-R1 plasmid pKN1562. Horizontal and vertical projections correspond respectively to the basic replicon module and the *kis-kid* antitoxin-toxin system. The relative positions of key genes in these modules and the promoters involved in RNA synthesis are also indicated.

CopB is a tetrameric repressor protein that inhibits transcription of *repA* from the internal and strong promoter *Prep*. The levels of this protein, that is transcribed from a constitutive promoter, decrease when plasmid copy number is reduced; at a very low copy number, the tetramer disassembles and the protein lose activity as a repressor; this increases transcription of *repA* and as consequence the copy number of the plasmid increases. In this way, *copB* acts as a proper copy number control gene that contributes by rescuing inefficient plasmid replication [6,7,8]. Once the plasmid copy number is restored, CopB levels increase and the protein multimerizes and recovers its repressor activity. Increasing CopB levels *in trans* favours CopB repressor function but removes its potential to rescue very low plasmid copy number [9]. *copB*, *copA*, *copT*, *repA* and *oriR1*, the so called “basic replicon” [10], is the essential maintenance module of the plasmid.

*Kis-kid* or *parD* is an auxiliary maintenance module of R1 that is close to the basic replicon [11,12] (see Figure 1). This system contains two genes, *kis* and *kid*, encoding respectively two small proteins: an antitoxin, Kis (killer suppressor), and a toxin, Kid (killing determinant)*.* Kid is an RNase that cleaves RNA at sites containing the core sequence 5'-UA(A/C/U)-3; flanking U increase the efficiency of cleavage at these core sequences [13,14,15]. Kis, the antitoxin, is a protein that interacts with Kid and neutralizes its activity. Kis is also a specific repressor of the operon whose efficiency increases in complex with Kid [16,17].

*kis-kid* activity is functionally coupled to the efficiency of R1 replication. The first indication of this coupling was indirect: *kis-kid* interfered with the isolation of plasmid replication mutants; this was due to the activity of Kid: mutations that inactivated the Kid toxin abolished this interference [18]. Since then, this phenotype, called the “interference” phenotype, has been used as one of the signatures of this coupling. It was later reported that the *kis-kid* system is activated in low copy mutants of the plasmid and that this partially recovered the plasmid copy number [19]. Two findings were key to explaining, in molecular terms, this new and intriguing signature of the coupling: (i) the identification of the RNase activity of the Kid toxin [13,14,15] and (ii) the finding that *copB-repA* mRNA contains two sites in the intergenic region of *copB-repA* mRNA that are efficiently cleaved by Kid [20]; this cleavage reduces the CopB levels, activates the *repA* promoter and increases plasmid replication efficiency. It has been recently reported that Kid cleaves mRNA of key cell division proteins; in this instance, Kid replication rescue occurs before cell division and effectively enforces plasmid retention by uncoupling plasmid replication and cell division [21]. We reported recently that increasing *in trans* the Kis antitoxin levels suppressed the “interference” phenotype; this suggested that Kis antitoxin levels could act as the switch connecting the replication and *kis-kid* toxin-antitoxin maintenance functions [22]. Coupling between replication functions and other maintenance modules has been reported in other plasmids systems: in pSM19035 of *Streptococcus pyogenes* a global regulator couples plasmid replication, partitioning and toxin-antitoxin modules to achieve high plasmid stability [23]; in the broad host range plasmid RK2, a global regulator controls expression of replication and maintenance systems in different hosts [24]. In the repABC plasmid family of Rhizobiales, transcription of the gene of the replication initiation protein is controlled by proteins of the plasmid partitioning system [25]. In ColE1 plasmid, a multimer resolution system, XerCD couples replication and cell division to achieve plasmid maintenance [26] (see Discussion).

The results reported here support the role of Kis antitoxin as the switch that couples replication functions and the kis-kid maintenance system; they also support the proposal that a Kid-dependent decrease in the CopB levels increases plasmid replication efficiency. In addition, we found that, beyond playing a role as a replication safety device, coupling between plasmid replication and the *kis-kid* system increases significantly the copy number of the wt plasmid R1. This implies that this coupling plays a significant role as part of the basic mechanisms that control plasmid R1 replication.

## 2. Results

### 2.1. Kis Antitoxin Levels Decrease in Cells Containing a pKN1562 Replication Mutant and Increase in a clpP^−^ Background

**Figure 2:**
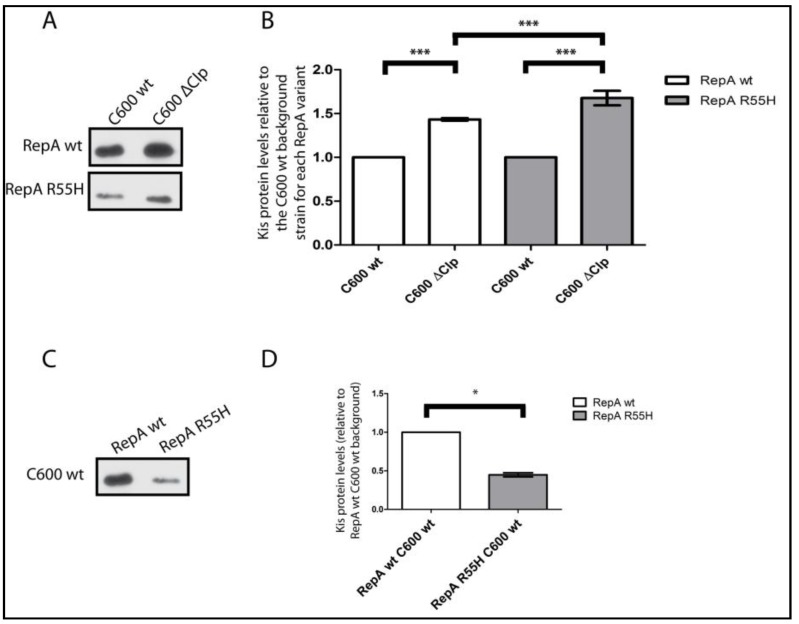
Inmunodetection of Kis antitoxin levels. Western blot (**A**,**C**) and densitometric analysis (**B**,**D**) of Kis levels determined in C600 or in its *clpP^−^* derivative (C600−∆*clpP*). Cells contained either the pKN1562 mini-R1 plasmid coding for RepAwt or its *repA55* mutant. The *repA55* mutation changes R for H in codon 55 of RepA and this results in a thermosensitive replication protein that, at the permissive temperature, reduces plasmid copy number. An equal amount of total protein extracts were loaded in each lane of the gels and the inmuno-signal of the 3×FLAG labeled Kis was densitometred. Values in B and D represent the average of seven independent densitometries and are corrected for the number of plasmid-containing cells. This percentage was 100% for cells containing the wt plasmid and 78% for cells containing the *repA55* plasmid replication mutant. * or *** indicate differences which *p*-values are = 0.01–0.05 or <0.001 respectively.

To evaluate the proposal that a reduction in the efficiency of plasmid R1 replication reduced the Kis levels, we compared these levels in cells containing plasmid pKN1562 or its *repA55* mutant. This mutation reduces significantly the copy number of the plasmid and activates the modular coupling (see Section 2.2). For these determinations, we tagged *kis* antitoxin genes of the wt and *rep* mutant plasmids with a 3×FLAG epitope that is recognized efficiently by specific monoclonal antibodies. Kis antitoxin levels were subsequently evaluated by inmuno-blotting. The data (Figure 2C,D) show that the Kis antitoxin levels decreased in cells containing the plasmid-*repA* mutant. Consistently with the role of ClpAP as the specific protease cleaving Kis [27], the levels of this protein increased in a strain carrying a deletion of the gene of the ClpP protease; this increase occurred in cells containing the *repA-wt* miniR1 plasmid pKN1562 or its *repA55* mutant (Figure 2A,B).

### 2.2. Effects of Increasing the Stability of the Kis Antitoxin on the Modular Coupling

Coupling between replication functions and the *kis-kid* system reduces drastically the frequency of isolation of plasmid replication mutants (interference phenotype). This phenotype, a signature of the modular coupling, can be abolished by inactivating the gene of the Kid toxin or by overproducing the Kis antitoxin [22]. We now tested if the increased levels of the Kis antitoxin shown in Figure 2 associated to its stabilization in a *clpP^−^* strain abolished this phenotype. To this aim we transformed C600 and its *clpP*^−^ mutant with a preparation of pKN1562 mutagenized with hydroxylamine. The number of pKN1562 thermo-sensitive replication mutants (*rep-ts*) recovered in this transformation was compared with the number of kanamycin resistant thermo-sensitive mutants (*kmr-ts*) rescued in the same screening. Note that the rescue of kmr-ts mutants is independent of the presence of a *kis-kid* system; therefore, they serve as a reference to determine the relative number of *rep-ts* mutants isolated in different conditions [22]. The analysis indicated that the *rep-ts/kmr-ts* ratios were 0.22 in the wild-type strain (2/9) and increased to 1.1 in the *clpP^−^* strain (10/9). The increase ratio obtained in this strain indicates that stabilization of Kis antitoxin inactivates the interference phenotype, which implies inactivation of the modular coupling. Note that a similar increase ratio has been reproduced in experiments in which the modular coupling was inactivated either by inactivation of *kid* or by overproduction of Kis [22]. The experimental procedures are detailed in this reference and these procedures are only briefly discussed here (material and methods).

**Figure 3:**
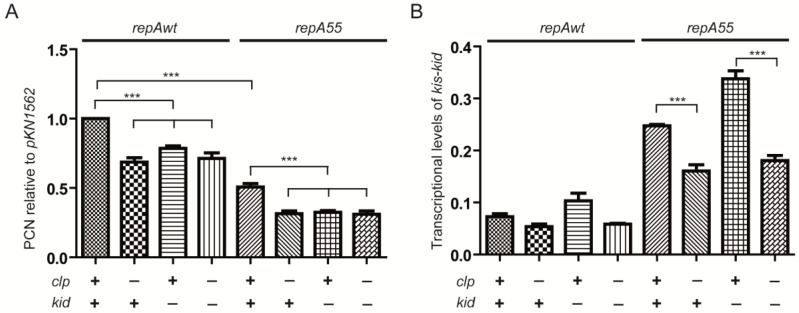
Effects of stabilizing Kis antitoxin (**A**) on plasmid replication and (**B**) on *kis-kid* transcriptional levels. Analyses were done in a wt strain or in an isogenic *clpP^−^* background using plasmids KN1562 and its *repA55* thermo-sensitive replication mutant; *kid* + and *kid* − indicates respectively analysis carried out in the presence of a pKN1562 or of its *kid75* mutant pJLV01. (**A**) Plasmid copy number determinations (PCN) of pKN1562 (*kid* +) and its *kid75* mutant (*kid* −) in the presence/absence of a functional *clpP* gene (*clp* + or *clp* −). Values were corrected by the number of plasmid free cells and referred to PCN of the *lpp* chromosomal gene (see Materials and Methods). The PCN value corresponding to pKN1562 in the wt strain (*clp* +, *kid* +) was given the reference value of 1; (**B**) Transcriptional levels of *kis-kid* per plasmid copy corresponding to the samples analyzed in panel (**A**). Transcriptional levels of the *lpp* gene were used as the reference. *** indicates differences which *p*-values are <0.001.

A second signature of the coupling is the replication rescue phenotype, meaning a partial recovery of the efficiency of plasmid replication of *repA* mutants mediated by *kis-kid*. Data in Figure 3A show that in a wt background (*clp* +), the *repA55* mutation reduced significantly the copy number of pKN1562. In the isogenic *clpP^−^* strain (*clp* −), this value is further reduced. The difference found is statistically significant and indicates that stabilization of the Kis antitoxin, protein in this last strain abolishes the replication enhancement effect dependent on *kis-kid*. The results are consistent with the proposal that the *clpP^−^* background that stabilizes the Kis antitoxin, inactivates the modular coupling. As a control, it is shown that inactivation of the coupling due to the *kid75* mutation, abolishes the replication rescue effect. Important, during this analysis we realized that in the *clpP^−^* background, the copy number of the wt plasmid pKN1562 is significantly reduced. Furthermore, the *kid75* mutation that inactivates the modular coupling reduces the efficiency of plasmid wt replication to a similar level. This result revealed that the communication between the two maintenance modules form part of the basic mechanisms that control plasmid R1 replication. Results presented in the next two sections further support this conclusion.

Coupling between replication and *kis-kid* modules is also associated with a de-repression of the *kis-kid* operon. To follow this correlation, we determined the *kis-kid* mRNA levels in cells containing the wild-type pKN1562 plasmid or its *repA55* mutant (Figure 3B). An increase in *kis-kid* transcription level is most clearly observed in the *repA55* mutant and in the presence of a wt *clpP* gene (*clp* +). In the absence of *ClpP* (*clp* −), Kis repressor levels are stabilized, and accordingly, transcriptional levels of *kis-kid* are significantly reduced. In cells containing plasmid pKN1562 wt (kid +), *kis-kid* transcription is maintained at a basal level.

Note that the *kid75* mutation inactivates the modular coupling as it inactivates the RNase activity of the toxin; however, the mutations do not interfere with the co-regulatory potential of the toxin and this allows evaluation of *kid* mutation on *kis-kid* transcription. We noticed that the presence of the *kid75* mutation significantly increases *kis-kid* transcription levels implying a negative effect of the RNase activity of Kid on the levels of the *kis-kid* transcript.

### 2.3. Effects of Overproducing the Kis Antitoxin on the Modular Coupling

Previous results indicate that overproduction of the Kis antitoxin abolished the interference phenotype meaning inactivation of the coupling [22]. We now completed the analysis testing the effect of this overproduction on the replication rescue phenotype and on the transcriptional level of the *kis-kid* operon.

**Figure 4:**
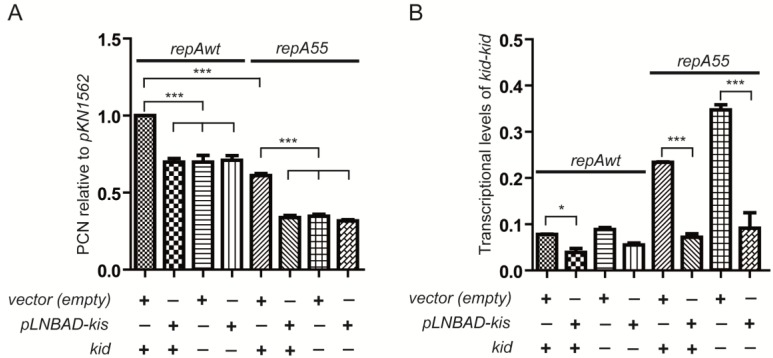
Effects of overproducing Kis antitoxin on the modular coupling. pLNBAD-kis is a conditional overproducer of the Kis antitoxin and + or − indicates respectively presence or absence of inducer (arabinose). As a control, analyses were carried also in the presence of the empty vector (pLNBAD). Effects on KN1562 wt, its *repA55* mutant and on derivatives of these plasmids carrying the *kid17* mutation were determined. (**A**) Copy number determinations. The PCN value corresponding to pKN1562 was given the reference value of 1; (**B**) *kis-kid* transcriptional levels. Values were corrected by the number of plasmid free cells and referred to the PCN or the transcriptional level of the *lpp* chromosomal gene (see Materials and Methods). * or *** indicate differences which *p*-values are = 0.01–0.05 or <0.001 respectively.

As activation of the modular coupling can also be detected in cells containing the wild-type plasmid pKN1562 (2.1.), we first evaluated the replication rescue phenotype testing the copy number of this plasmid in the presence or absence of excess of Kis antitoxin; this excess was provided *in trans* by the Kis overproducer pMLM126 in the presence of inducer. Control values were obtained in the presence of the empty vector pLNMBAD. Data show that indeed, the copy number of pKN1562 is substantially reduced in excess of Kis antitoxin (Figure 4A). This result confirms the effect of the coupling on the basal efficiency of replication of pKN1562 and implies that excess of Kis antitoxin inactivates the coupling. Data in Figure 4A further show that the *kid75* mutation (pJLV01) abolishes the replication rescue effect, which is consistent with the dependence of this phenotype on an active toxin.

Similar conclusions can be obtained when a pKN1562*repA55* mutant is used. As expected the *repA55* mutation significantly reduces plasmid copy number. Again the *kid75* mutation further reduces the copy number of the *repA55* mutant. This reduction is similar to the one observed in excess of Kis meaning that in both cases the modular coupling is lost.

Figure 4B shows the effect of excess of Kis on *kis-kid* mRNA levels determined in the same conditions analyzed in 4A. The results, best shown in the presence of the *repA55* mutation, indicate that this mutation de-represses the *kis-kid* operon and that de-repression is substantially neutralized in excess of the Kis antitoxin. Although to a lower level, the effect is also seen for the pKN1562 wt plasmid. Note that, as previously shown (Figure 3B), the *kis-kid* mRNA levels increase in the presence of the *kid75* mutation (*kid*−).

The above results confirm that an excess of Kis abolishes the coupling between plasmid replication and *kis-kid* maintenance systems both in the wt plasmid and in its *repA55* mutant.

### 2.4. Coupling between Maintenance Modules and Plasmid Stability

**Figure 5:**
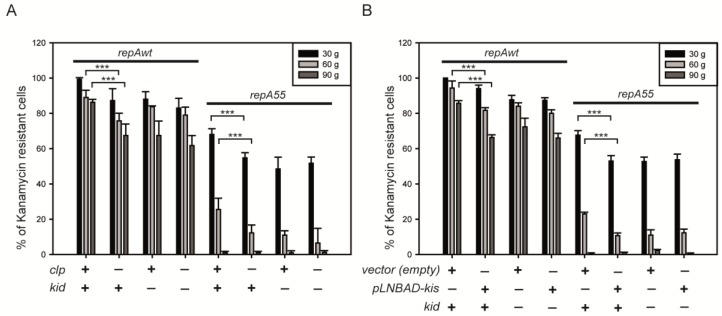
Effects of abolishing the coupling between maintenance modules on plasmid stability. Plasmid stability was followed determining the percentage of cells retaining the kanamycin resistance marker of pKN1562 and its derivatives after propagation at 30 °C for 30, 60 and 90 generations in rich non-selective media (see Experimental Section). Panel (**A**) shows the effect of the *clpP^−^* background on plasmid stability and Panel (**B**) shows the effect of overproducing the Kis antitoxin. *** indicate differences which *p*-values are <0.001.

Modular coupling influences plasmid copy number and this has a direct effect on plasmid stability. We evaluated this correlation studying plasmid stability in the presence or absence of a mutation in the host that stabilizes the Kis antitoxin (clp – or clp +) or in the presence or absence of an excess of Kis antitoxin (pLNBAD-kis + or −). As shown previously, both conditions inactivate the modular coupling. In the analysis we used the same strains and constructions used for copy number determinations (see Section 2.2. and Section 2.3.). The results (Figure 5) show that there is a correlation between plasmid copy number and plasmid stability. The effects on plasmid stability are seen more clearly after 60 or 90 generations of propagation in non-selective medium. Conditions that abolish the modular coupling reduce plasmid copy number and this reduces plasmid stability. As predicted, the *repA55* mutation that reduces the efficiency of plasmid replication results in all cases in less stable plasmids.

The results of the stability analysis are consistent with the role of the coupling between maintenance modules in plasmid copy number and are also consistent with the proposal that Kis antitoxin is the switch that connects these modules.

### 2.5. Excess of CopB Abolishes Replication Rescue

**Figure 6:**
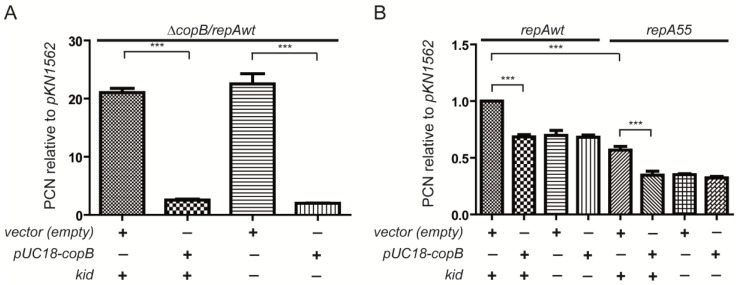
Effects of an excess of the CopB protein on the efficiency of replication of mini-R1 plasmids. (**A**) PCN of *copB* mutants of pKN1562 (kid +) and of its *kid75* mutant (kid −) determined in the presence of and overproducer of CopB (pUC18-*copB*) or of its empty vector (pUC18); (**B**) Relative PCN values of the wt pKN1562 plasmid and its *repA55* mutant. Values were corrected by the number of plasmid free cells and referred to the PCN of the *lpp* chromosomal gene (see Materials and Methods). Determinations in the presence (+) or absence (−) of CopB overproducer or of its pUC18 vector were done in the presence of a wt *kis-kid* system (kid +) or of its *kid75* mutant (kid −). In (**A**) and (**B**), the PCN value corresponding to pKN1562 in the presence of the empty vector (pUC18) was given the reference value of value 1 (A and B). *** indicate differences which *p*-values are <0.001.

The replication rescue phenotype is dependent on the cleavage of the *copB-repA* mRNA mediated by the RNase activity of the Kid toxin [20]. We aimed to test the effects of an excess of CopB protein on this phenotype. The analysis was done in the presence or absence of the *kid75* mutation both in the pKN1562 wt plasmid and in its *repA55* mutant. As an exogenous source of CopB, we used a multi-copy pUC18-*copB* recombinant that greatly increases the levels of CopB [7]. The functional effect of this excess can be monitored evaluating the complementation of mini-R1 *copB* deletion mutation that removes the *copB* promoter and part of the *copB* gene, thus increasing the plasmid copy number. The results (Figure 6A) show that in the presence of the pUC18-*copB* recombinant, the copy number of *copB* deletion mutants of pKN1562 or of its *kid75* mutant pJLV01 decreases from values close to 20–25 to a basal level. This clearly indicates that the excess of CopB provided *in trans* complements the *copB* mutation. The controls made using the pUC18 vector alone indicates that the complementation observed is a specific effect of CopB.

We then tested the effect of an excess of CopB on the replication efficiency of plasmids pKN1562 or pJLV01, both of them carrying a wt *copB* gene and therefore a low copy number. Not that pJLV01 carries the *kid75* mutation that inactivates the modular coupling. Data in Figure 6B show that, in the presence of the pUC18-*copB* recombinant, the copy number of pKN1562 is significantly reduced to the value corresponding to pJLV01. This result indicates that the replication rescue is abolished in excess CopB. As expected, the *repA55* mutant of pKN1562 has a lower copy number than the wt plasmid; similarly, excess of CopB reduces the plasmid copy number of this *repA* mutant and this reduction is similar to the one observed in the *repA* mutant carrying the *kid75* mutation.

These data are consistent with the proposal that the replication rescue associated to the modular coupling is dependent on a reduction of the CopB levels due to the action of the RNase activity of Kid toxin. The results confirm again that the modular coupling form part of the basic mechanisms that control plasmid R1 wt replication.

## 3. Discussion

### 3.1. Modular Coupling between Maintenance Modules and Efficiency of Plasmid wt Replication

The results of this work consistently support the model for the role of Kis antitoxin as the switch that connects the replication and *kis-kid* toxin-antitoxin modules of the plasmid. The Kis antitoxin levels are clearly reduced when copy number of the plasmid, that activates the coupling, is reduced. Conversely, it is shown that stabilizing the Kis antitoxin or increasing its levels prevented the modular coupling. This coupling was assessed testing the interference and replication rescue phenotypes as well as studying the transcriptional levels of the operon. Modular coupling and effective replication rescue requires normal levels of CopB. The analyses also show that increasing the CopB levels or inactivating the Kid toxin prevents the modular coupling. Interestingly, we found that the different ways of uncoupling plasmid replication and *kis-kid* modules consistently reduce plasmid copy number not only of the *repA55* mutant but also of the wt plasmid. This implies a more direct involvement of this coupling in control of plasmid replication. The plasmid copy number distribution in individual cells of the culture can reach a significant part of the population, the low level required to trigger the coupling (discussed in [9]). Improving copy number or partitioning at cell division has been pointed out to explain the unexpected high stability of low-copy plasmids [28]. It has been pointed recently that Kid can inhibit cell division and that this toxin achieves plasmid retention by uncoupling plasmid replication and cell division [21]; in addition, the same study proposes that the *hok-sok* antitoxin-toxin system of this plasmid could eliminate plasmid free cells that could arise due to failures in this process.

### 3.2. The Pathway: Kis Antitoxin Acts as the Switch Connecting Plasmid Replication and Toxin-Antitoxin Modules

The analysis reported here indicate that the coupling between the basic replicon of R1 and the *kis-kid* system is initiated when the copy number of the plasmid falls; this reduces the antitoxin level and activates the potential of the Kid toxin to induce the “interference” or the “replication rescue” phenotypes. This last phenotype is dependent on *kid* and *copB*; it is activated in cells containing the wt plasmid or its replication mutants and is well defined in molecular terms. The interference phenotype is a signature of the molecular coupling and as the replication rescue is dependent on a wt Kid toxin but it is poorly defined in molecular terms.

### 3.3. On the Inhibition of the Replication Rescue by Excess of CopB

The replication rescue phenotype is a late event in the pathway described above. It is dependent on Kid and CopB activities. Overproduction of CopB abolishes this phenotype but in principle should not affect the regulation of the *kis-kid* module as the sequence targeted by this transcriptional repressor is not present in the promoter of the *kis-kid* operon. In addition CopB overproduction should not affect the “interference” phenotype as CopB does not affect the activity of Kid required for this phenotype. When we compared the plasmid copy number obtained in the presence or absence of an excess of CopB we observed a significant replication enhancement in the presence of normal levels of CopB that was abolished in excess of CopB. This effect is measurable and it can be assigned to the molecular coupling described here.

The modular coupling forms part of the auxiliary machinery that controls plasmid R1 replication via CopB. Its action depends on a reduction of the antitoxin levels that activates the Kid toxin and this can be affected by plasmid copy number but also by the ClpAP protease. Thus the activity of these effectors can influence the role of CopB as auxiliary copy number control protein.

### 3.4. Coordination of Plasmid Maintenance Functions in Different Systems

Growing information underlines the relevance of connections replication and plasmid maintenance modules and eventually with cell cycle events in different plasmids. In pSM19035, a plasmid of *Streptococcus pyogenes*, replication, partition and toxin-antitoxin systems act co-ordinately to achieve high plasmid maintenance level with a minimal fitness cost [23]. In the broad host-range plasmid, RK2 replication and partitioning functions act co-ordinately to achieve stable plasmid maintenance in different hosts. Multiple co-ordinately regulated operons contribute to this [24] and the basic mechanisms involved have been recently reviewed [29]. Other singular case of coordination between different functions related to plasmid maintenance has been reported in the XerCD multimer resolution system of plasmid ColE1. Multimers of this multicopy plasmid compromise cell growth and plasmid stability but they can be resolved by the XerCD-mediated site-specific recombination at *cer* [30]. In addition, multimer formation induces *rcd*-RNA, a singular component of the system that interacts with and enhances the action of triptophanase thus increasing the concentration of Indol in the cells. This inhibits cell growth and division as well as plasmid replication, thus timing the resolvase to act before cell division can occur [31]. Similarly, in plasmid R1, coordination of the basic replicon functions and the *kis-kid* system activates the Kid toxin to rescue replication of cells with very low copy number [16]. Activated Kid toxin inhibit cell division, thus effectively achieving plasmid R1 retention by increasing plasmid replication and timing this replication rescue before cell division can occur [21]. Our report adds to this that the *kis-kid* system plays a more relevant role in control of plasmid copy number than previously suspected.

## 4. Experimental Section

### 4.1. Cell Cultures, Strains and Plasmids

Cells were growth at 30 °C in L-Broth (LB) and L-Broth agar (LA) prepared as described [32]. Antibiotics were supplemented according to the resistances carried by the plasmids. The *E. coli* K12 *clpP^−^* mutant SG12050 [33] and its parental strain C600 [34] were used in this work. Plasmid used in this work were: pKN1562, a wild-type (wt) mini-R1 plasmid conferring resistance to kanamycin [10] and carrying the *wt kis-kid* system [11]; pJLV01, a pKN1562 derivative that carries the point mutation *kidD75E* that inactivates the RNase function of the Kid toxin but not its co-regulatory activity [22,35,36]; variants of pKN1562 or pJLV01 carrying the *repAR55H* mutation [22]; pMLM126, a Kis overproducer inducible by arabinose, and pLNBAD its empty vector [36]; finally, we also used a CopB overproducer, pUC18-*copB*, and its empty vector pUC18 [6].

### 4.2. Replication Interference Phenotype

Plasmid DNA extractions and transformations were essentially as described [37]. *In vitro* mutagenesis of plasmids was done with hydroxylamine as previously described [38]. This DNA was extensively dialysed against TE buffer (Tris-HCl 10 mM pH. 8.0, 1 mM EDTA) and used to transform the selected *E. coli* strains. The ratio of replication thermo-sensitive mutants (*rep-ts*) to kanamycin resistant thermo-sensitive mutants (*kmR-ts*) rescued by transformation was used to define the replication interference phenotype. This value is very close to 1 when the coupling is inactivated by a mutation in the *kid* toxin gene but it is reduced to 0.2–0.3 in the presence of a functional coupling [22].

### 4.3. Plasmid Stability and Statistical Analysis

The plasmid stability determinations were done in cells containing pKN1562 and its *rep* and *kid* mutant derivatives. For this purpose the percentage of colonies carrying the kanamycin resistance marker were determined after cell propagation at 30 °C for 30, 60 and 90 generations in rich solid LA media without kanamycin as previously described [22].

The standard deviation corresponding to the different determinations, were calculated using values obtained at least in three independent experiments. A paired Student’s *t*-test using GraphPad Prism 5 for Mac software was used. For the statistical analysis of Kis antitoxin levels One way ANOVA test and Wilcoxon non-parametric *t*-test were used. Values linked by brackets and labelled with * or *** correspond to differences which *p*-values are <0.05 or <001, respectively.

### 4.4. Plasmid Copy Number Determinations

R1 mini plasmids copy numbers relative to chromosome were determined by quantitative PCR methodologies (qPCR) as described in [39]. In these determinations, *kis* and *lpp* genes were selected as plasmid and chromosome markers, respectively. Specific protocols and primers used in these determinations have been described recently [22]. Plasmid copy number (PCN) per genome was calculated as the ratio between (Size of chromosomal DNA (bp) × Amount of plasmid DNA (ng))/(Size of plasmid DNA (bp) × Amount of genomic DNA bound (ng)). The PCN calculated for pKN1562 was giving the value = 1; the PCNs per genome were normalized to this value. When the analysis was done in the presence of the overproducer of Kis, the primers used for the PCN and transcriptional determinations amplified the 5'region of the Kid toxin and were 5'-GGTCACGCGATTAAAGGC-3' and 5'-GGTCACGCGATTAAAGGC-3'. The PCN values were referred to as the percentage of kanamycin resistant cells evaluated when the sample was taken.

### 4.5. Quantification of KIS-KID MRNA LEVELS

Transcriptional levels of *kis* and *lpp* genes were evaluated by RTqPCR [22]. The *kis* transcriptional levels were calculated as 2^(Ctlpp−Ctkis)^ where C_t-_*lpp* and C_t-_*kis* are the threshold values corresponding to the PCR amplification of *lpp* and *kis*. When the analysis was done in the presence of the Kis overproducer, the primers used for the *kis-kid* mRNA determinations (see Section 4.4) amplified the 5' region of *kid* (see Section 4.4). The relative transcriptional levels were referred in all cases to the plasmid copy number, a value that is already corrected from the contribution of plasmid-free cells.

### 4.6. Determination of Kis Antitoxin Levels by Western Blotting-3XFLAG

Bacterial cultures were growth as indicated above. Equal samples of the cultures were taken at DO_600_ = 0.5 and the cells were collected and re-suspend in Laemmli buffer. The number of plasmid-containing cells was evaluated in these samples. Antitoxin expression levels were determined as the means of at least seven Western blotting experiments using a pKN1562 version with 3×FLAG-tagged Kis. Kis protein values were referred to the percentage of plasmid-containing cells; the average level determined in the wild-type strain was used as the reference (value = 1). Western blot analysis was done using anti-FLAG (Sigma-Aldrich, St. Louis, MO, USA) diluted 1:500 (2 h 30 min) and anti-mouse antibodies (Sigma-Aldrich, St. Louis, MO, USA) diluted 1:5000 (1 h 30 min) in TBS-Tween containing 3% non-fat milk. The westerns were developed using ECL (Thermo-Scientific, Waltham, MA, USA); band intensity analysis corresponding to samples from five independent cultures, was determined using Quantity One software (Bio-Rad, Berkeley, CA, USA, version 4.6.3).
